# Tumoral Calcinosis of Thoracic Spine Associated with Vertebral Fracture and Inflammatory Reactions

**DOI:** 10.1155/2020/8881698

**Published:** 2020-07-24

**Authors:** Isamu Miura, Motoo Kubota, Kento Takebayashi, Oji Momosaki, Koichi Honma, Takakazu Kawamata, Masahito Yuzurihara

**Affiliations:** ^1^Department of Spinal Surgery, Kameda Medical Center, 929 Higashi-cho, Kamogawa-shi, Chiba 296-8602, Japan; ^2^Department of Neurosurgery, Tokyo Women's Medical University, 8-1 Kawada-cho, Shinjuku-ku, Tokyo 162-8666, Japan; ^3^Department of Pathology, Kameda Medical Center, 929 Higashi-cho, Kamogawa-shi, Chiba 296-8602, Japan

## Abstract

Tumoral calcinosis involving the spine is rare. The involvement of the thoracic spine is rarer than that of the cervical or lumbar spine. We report a case of thoracic tumoral calcinosis accompanied by vertebral fracture with increased concentrations of inflammatory markers and no abnormalities in serum calcinosis and phosphorus concentration. A 60-year-old woman presented with complete paraplegia. Her white blood cell count and C-reactive protein (CRP) concentration were elevated. The thoracic magnetic resonance imaging revealed vertebral fracture and an epidural mass that demonstrated low intensity on both T2- and T1-weighted images at the T9/10 dorsal side of the central canal. This lesion is larger in size than that observed in the previous 2 months. Her laboratory data showed signs of infection, and only decompression surgery without fixation for treatment and diagnosis was performed. Histopathological examination was consistent with tumoral calcinosis. Postoperatively, the patient's white cell count and CRP concentration were normalized. We found that tumoral calcinosis can occur at the thoracic level on the basis of the spinal instability due to the vertebral compression fracture and the accompanying increase in inflammation indicated by increased white blood cell count and CRP concentration.

## 1. Introduction

Tumoral calcinosis is a pathologic entity characterized by periarticular masses caused by dystrophic calcification in the soft tissue. Tumoral calcinosis involving the spine is rare [1]. Moreover, the majority of cases of tumoral calcinosis involving the spine are either cervical or lumbar. Involvement of the thoracic spine is rare because spinal tumoral calcinosis may be common in this location as a consequence of degenerative changes. In general, the thoracic spine experiences less degenerative change compared with the cervical and lumbar spine [1]. Herein, we report a case of thoracic tumoral calcinosis accompanied by a vertebral fracture with increased concentration of inflammation marker and no abnormalities in serum calcinosis and phosphorus concentrations.

## 2. Case Report

**Figure 1:**
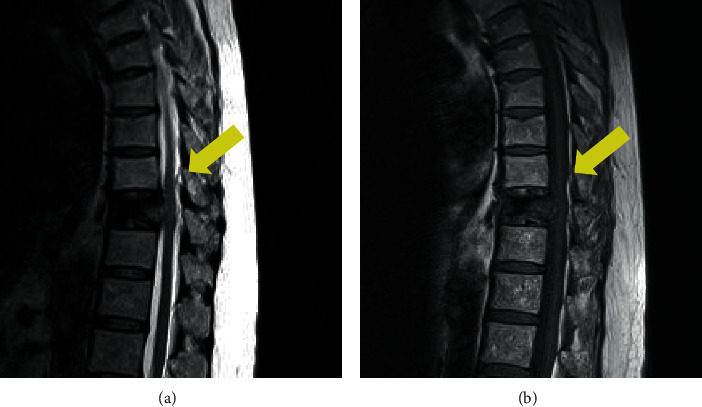
Sagittal views of thoracic magnetic resonance images on T2-weighted images (a) and T1-weighted images (b). T10 vertebral body demonstrated low intensity on the T2- and T1-weighted images and small epidural mass (yellow arrow) at the T9 dorsal side of the central canal was hyperintense on the T2-weighted images and was iso- to hyperintense on the T1-weighted images. These findings indicate T10 vertebral fracture and epidural hemorrhage.

**Figure 2:**
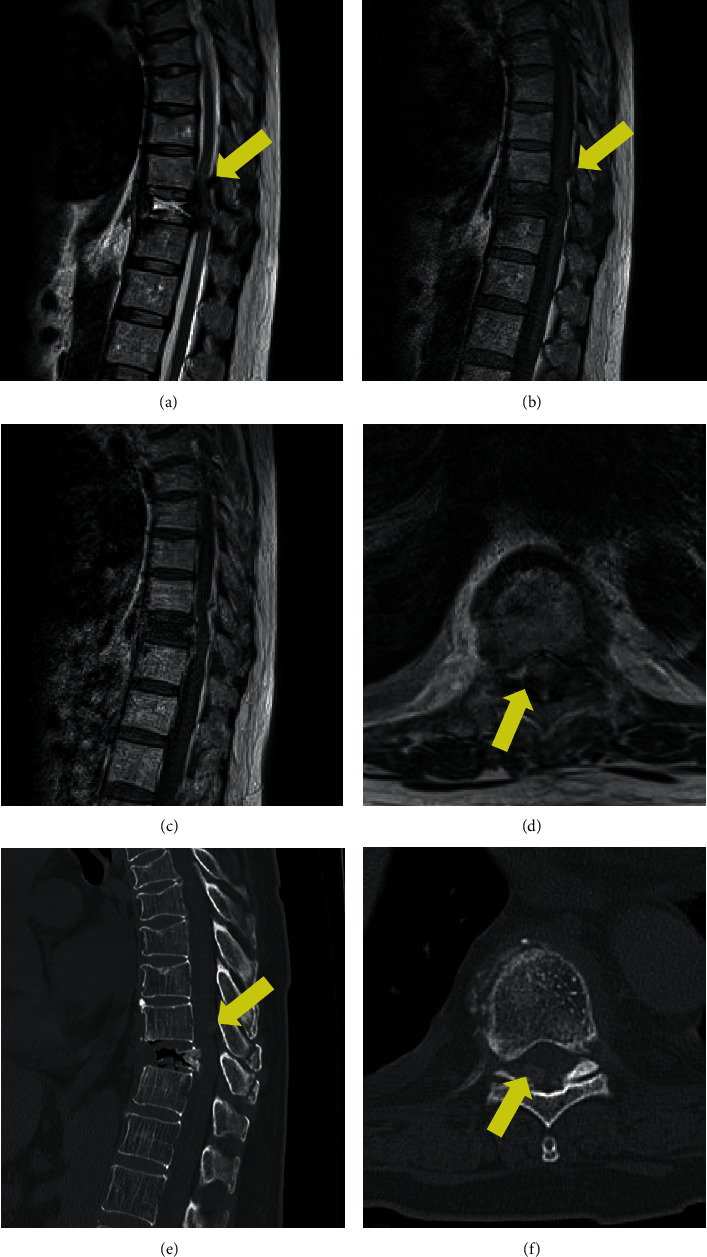
(a) T2 sagittal image showing a growing extradural mass (yellow arrow) at T9 level. (b) T1 sagittal image. (c) T1-enhanced sagittal image. (d) T2 axial image. (e) Computed tomography (CT) sagittal image demonstrating burst fracture of T10 vertebral body and slightly high-intensity mass at T9 level (yellow arrow). (f) CT axial image demonstrating mass effect at the right facet joint (yellow arrow).

**Figure 3:**
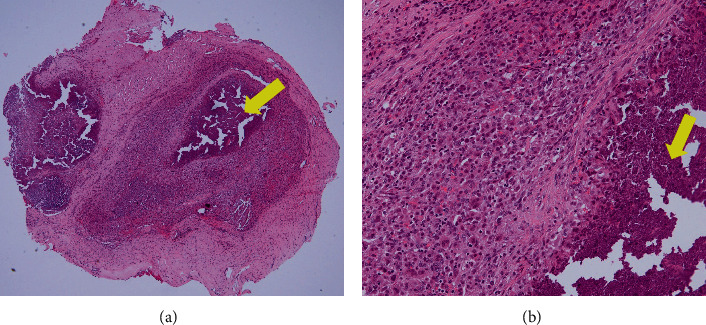
Hematoxylin and eosin staining. (a) Low power view (4x magnification). (b) High power view (20x magnification). Sections of the obtained specimen reveal multifocal massive deposition of calcified amorphous material (yellow arrow), which is surrounded by a thick layer of foreign body granuloma.

A 60-year-old woman had back pain with no neurological deficits. She had no history of fall or any other trauma. She had a medical history of hypertension, dyslipidemia, and benzodiazepine and alcohol dependence. She had no familial history. She was admitted to our hospital for further examination because her white blood cell count and C-reactive protein (CRP) concentration were more than 10,400/mm^3^ and 10.7 mg/dL, respectively. Chest and abdominal computed tomography (CT) did not reveal any lesion that caused inflammation. On the thoracic magnetic resonance imaging (MRI), the T10 vertebral body demonstrated low intensity on both T2- and T1-weighted images (Figures 1(a) and 1(b)). The MRI also revealed a small epidural mass at the T9 dorsal side of the central canal. The lesion was hyperintense on T2-weighted images and was isointense to hyperintense on T1-weighted images. Her diagnosis was established as T10 vertebral fracture and epidural hemorrhage, and conservative therapy was initiated. The patient's laboratory results showed improvement without antibiotic therapy. She was discharged 15 days after the hospitalization. However, 2 months after being discharged, her back pain was worsened after a fall. A few days after this event, she developed leg paralysis and visited another hospital. She was transferred again to our hospital for surgical treatment. A physical examination revealed her body temperature to be 36.7°C and her blood pressure to be 172/102 mmHg. Her heart rate was 119 in the sinus rhythm. Neurological examination showed complete paraplegia and numbness in the right and left lower limbs, which was evaluated as grade B on the American Spinal Injury Association (ASIA) Impairment Scale. Her white blood cell count and CRP concentration were 17,200/mm^3^ and 18.6 mg/dL, respectively. The calcium × phosphate product concentration in the blood was within the normal range. On the thoracic MRI, the T10 vertebral body demonstrated high intensity on T2-weighted images and low intensity on T1-weighted images (Figures 2(a) and 2(b)). The MRI also showed an epidural mass that demonstrated low intensity on both T2- and T1-weighted images at the T9 dorsal side of the central canal. This lesion had become larger in size than that observed previously. Contrast enhancement by the contrast medium in the T1-weighted MRI scan was not clear (Figure 2(c)). The spinal cord was compressed by this mass at this level (Figure 2(d)). CT demonstrated the T10 burst fracture and the cavity in the vertebral body (Figure 2(e)). The density of the epidural mass was slightly increased, and the mass was located near the T9/T10 facet joint (Figure 2(f)). Young adult means of bone mineral density measured by dual-energy X-ray absorptiometry at the lumbar spine and proximal femur were 93% and 85%, respectively. The patient was diagnosed with spinal injury caused by the T10 vertebral burst fracture and the compressive epidural mass. Her laboratory data showed signs of infection, and only decompression surgery without fixation for treatment and diagnosis was performed. T9–T11 laminectomy was performed. The presence of yellow ligament was confirmed; however, the epidural fat was unclear, which indicated inflammation. There was no obvious pus, and the soft tissue culture showed negative results. The epidural mass was attached to the dura matter. This mass was detached and removed. Histopathological examination of this mass revealed a multifocal massive deposition of calcified amorphous material (Figure 3), which was consistent with tumoral calcinosis. After the surgery, her neurological findings were improved to ASIA Impairment Scale grade C. Vancomycin and cefepime were administered postoperatively for 7 days. Her blood culture results were also negative, and her laboratory data were improved after the surgery. Thereafter, percutaneous transpedicular vertebral body biopsy was performed, and the fluid from the T10 vertebral body was collected and cultured; however, the results were negative, indicating no evidence of infection. Due to instability, fixation of the thoracic spine was required; however, the patient denied further surgery. After rehabilitation, she was able to stand and use a wheelchair with the help of an assistant.

## 3. Discussion

Most instances of tumoral calcinosis are found to occur in the shoulder, hip, and metatarsophalangeal joint. In a review by Smack et al., tumoral calcinosis was classified into the following three subtypes based on the pathogenesis [2, 3]:(1) primary normophosphatemic tumoral calcinosis without metabolic abnormalities, with no evidence of familial patterns [4], (2) primary hyperphosphatemic tumoral calcinosis with strong familial patterns, and (3) secondary tumoral calcinosis with concurrent disease capable of causing soft tissue calcification as chronic renal failure, hyperparathyroidism, hypervitaminosis D, scleroderma, pseudoxanthoma elasticum, malignancy, and milk–alkali syndrome [5]. Our patient had no familial history. She neither had renal failure nor abnormalities in calcium, phosphate, and vitamin D metabolism. We considered the case of this patient as primary normophosphatemic tumoral calcinosis.

Tumoral calcinosis is rarely located in the spine. Moreover, thoracic lesions are rarer than cervical and lumbar lesions [6]. Durant et al. reported only 3 cases of thoracic spine among 21 cases of spinal tumoral calcinosis. Spinal tumoral calcinosis may be common in this location as a result of degenerative changes [1]. It has also been reported that tumoral calcinosis occurs in the juxta-articular region [7, 8]. Synovial cysts are often considered a preoperative diagnosis because of the lesion location and the degenerative changes [3]. The degenerative change in the thoracic spine is generally less than that in the cervical and lumbar spine because of which thoracic lesions are rarer than cervical and lumbar lesions. Durant et al. reported that two of the three patients with thoracic lesions exhibited a vertebral compression fracture similar to our patient [1]. The vertebral compression fracture may cause spinal instability that may in turn result in the formation of spinal tumoral calcinosis in the facet joint.

**Figure 4:**
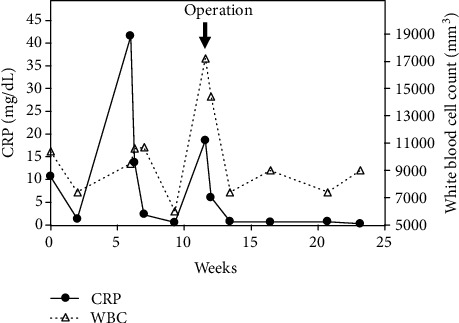
Time course of laboratory data of white blood cell count and C-reactive protein concentration. Arrow indicates the timing of surgical treatment.

In our patient, blood tests revealed an increased white blood cell count of 17,200/mm^3^ and a CRP concentration of 18.6 mg/L before the surgery. After the surgery, including the removal of tumoral calcinosis, the concentrations of these inflammatory markers were normalized (Figure 4). This implies that the formation of tumoral calcinosis may result in increased concentrations of inflammatory markers. Although there are some reports of tumoral calcinosis and inflammation, the majority of these cases were uremic tumoral calcinosis [9–12]. Uremic tumoral calcinosis occurring in patients with end-stage renal failure is a complication associated with high concentrations of serum calcium × phosphate product, and these patients often have secondary hyperparathyroidism. It has been reported that an increased calcium × phosphate product concentration is the major contributing factor to the development of tumoral calcinosis [11]. Uremic tumoral calcinosis is also associated with systemic inflammatory response. Conversely, Sebesta et al. reported a case of idiopathic tumoral calcinosis of the index finger associated with inflammatory response [13]. Laboratory investigations revealed an increased white cell count and CRP concentration with no abnormalities in serum calcinosis and phosphorus concentrations similar to our patient. In our patient, infection such as an epidural abscess was suspected because of the increased white cell count and CRP concentration. However, after the removal of the epidural mass, her white blood cell count and CRP concentration were normalized. This clinical time course indicated the relationship between inflammation and the formation of tumoral calcinosis.

In conclusion, we found that tumoral calcinosis can occur at the thoracic level based on the spinal instability caused by the vertebral compression fracture and the accompanying increase in inflammation indicated by an increased white blood cell count and CRP concentration. Therefore, tumoral calcinosis should be considered in the differential diagnosis of spinal cord compression, and it may be accompanied by inflammation that is indicated by an increased white blood cell count and CRP concentration.
